# Pancreatic Ductal Adenocarcinoma Induces Neural Injury that Promotes a Transcriptomic and Functional Repair Signature by Peripheral Neuroglia

**DOI:** 10.21203/rs.3.rs-2715023/v1

**Published:** 2023-03-28

**Authors:** Jonathan Weitz, Bharti Garg, Herve Tiriac, Alexei Martsinkovskiy, Sandip Patel, Andrew Lowy

**Affiliations:** University of California San Diego

## Abstract

Perineural invasion (PNI) is the phenomenon whereby cancer cells invade the space surrounding nerves. PNI occurs frequently in epithelial malignancies, but is especially characteristic of pancreatic ductal adenocarcinoma (PDAC). The presence of PNI portends an increased incidence of local recurrence, metastasis and poorer overall survival. While interactions between tumor cells and nerves have been investigated, the etiology and initiating cues for PNI development is not well understood. Here, we used digital spatial profiling to reveal changes in the transcriptome and to allow for a functional analysis of neural-supportive cell types present within the tumor-nerve microenvironment of PDAC during PNI. We found that hypertrophic tumor-associated nerves within PDAC express transcriptomic signals of nerve damage including programmed cell death, Schwann cell proliferation signaling pathways, as well as macrophage clearance of apoptotic cell debris by phagocytosis. Moreover, we identified that neural hypertrophic regions have increased local neuroglial cell proliferation which was tracked using EdU tumor labeling in KPC mice. This study reveals a common gene expression pattern that characterizes solid tumor-induced damage to local nerves. These data provide new insights into the pathobiology of the tumor-nerve microenvironment during PDAC as well as other gastrointestinal cancers.

## Introduction

The pancreas is innervated by sensory, parasympathetic, sympathetic fibers (1–3) and receives neuronal input from enteric neurons of the gut (4). A dramatic increase in neural density is observed in pancreatic ductal adenocarcinoma (PDAC) (5, 6). During perineural invasion (PNI), PDAC cells hijack and utilize pancreatic neural networks as conduits for metastasis. These paracrine signaling interactions between nerves and pancreatic cancer cells have been shown to promote cancer cell migration (7) and proliferation (6).

The presence of PNI has been shown to be a poor prognostic factor and is estimated to be found in 70– 100% of PDAC specimens (8). Currently, there are no approved treatments which target PNI. While nerve and cancer cell interactions have been the focus of most PNI research, the Schwann cell is the most abundant cell type found within nerve bundles (9). A recent report demonstrated that Schwann cells within pancreatic nerves organize into dynamic tracks that promote cancer cell migration and resemble non-myelinated repair-type Schwann cells (10). In other pathological conditions such as spinal injury, repair-Schwann cells align into Bünger bands which locally proliferate, enable axonal guidance, and promote nerve regeneration; reviewed in (11).

To better understand the functional and molecular signals regulating perineural invasion and the local tumor-nerve microenvironment, we performed digital spatial analysis to characterize changes occurring between thin caliber axon fibers, more commonly present during physiologic conditions in the pancreas, as well as larger nerve bundles, which are commonly found during PDAC. We show that large nerve bundle regions have transcriptional signatures characterized by the upregulation of BMP, MAPK and JUN signaling, as well as apoptosis. Additionally, we show that in PDAC, nerve associated macrophages express gene signatures indicating their role in phagocytosis. Moreover, these neuroglial actively proliferate within PDAC. This study provides further insights into the functional and molecular framework of the tumor-nerve microenvironment.

## Methods

Immunofluorescence and Confocal Imaging: KPC tumor tissues were fixed in 4% paraformaldehyde and subsequently cryoprotected (30% sucrose). Tissue sections were cut at (40 μm) cut on a cryostat. After permeabilization (PBS–Triton X-100 0.3%), sections were incubated in blocking solution (permeabilization buffer with 1% donkey serum). Primary antibodies were diluted in blocking solution. Immunofluorescence images were aquired using confocal microscopy using a Nikon Ti microscope with integrated autofocus, automated XY and Z stage, A1R hybrid confocal scanner including a highresolution (4096×4096) scanner, LU4 four-laser AOTF unit with 405, 488, 561, and 647 lasers, Plan Apo 10 (NA 0.8), 20X (NA 0.9) dry objectives. To visualize macrophages, we used antibodies against F4/80 (1:200, catalog number 123116; BioLegend, San Diego, CA; and 1:200 catalog number MCA497R; Bio Rad, Hercules, CA), PanCK to visualize epithelial cells (1:100, catalog number SC8018; Santa Cruz Biotechnology, Dallas, TX). Cell nuclei were stained with DAPI 1:1000 4′, 6-diamidino-2-phenylindole (DAPI) (Catalogue Number D1306; Thermo Fisher/Life Technologies, Waltham, MA). Slides were mounted with ProLong Antifade (Thermo Fisher/Life Technologies, Waltham, MA). H&E-stained slides were used for morphologic evaluation of tumors.

### GeoMx Nanostring and Bioinformatics analysis

GeoMx^®^ Digital Spatial Profiling (DSP) was performed using KPC mouse tissues from nerve bundles, nerve regions containing macrophages, nerve fibers, acinar, acinar tissue containing nerve bers and spleen for a total of 23 geometric regions of interest (ROIs). Morphology markers included antibodies for tyrosine hydroxylase, F4/80, PanCK, and and Syto 83 orange DNA dye to visualize tissue morphology guiding the selection of the 23 ROIs. Differential expression analysis, PCA plots, heatmaps and gene ontology analysis was generated using the BioJupies maayanlab.cloud pipeline (12). To eliminate any enrichment bias, sequential ROIs from KPC section were not included in analysis, and only included for validation controls.

### Tumor and neuroglia proliferation analysis

KPC mice were monitored daily until a palpable tumor was discovered. Subsequently, daily EdU injections (catalog number 900584; Sigma Aldrich, Burlington, MA) were given (50 mg/kg) until euthanasia. Tumors were removed, fixed in 4% PFA and cryoprotected in 30% sucrose. EdU was detected using the Click-iT EdU kit (Catalogue Number C10337; Thermo Fisher/Life Technologies, Waltham, MA). Colocalization analysis of confocal images of TH + and EdU + cells was performed using ImageJ.

### Human protein atlas tissues

Protein analysis on human tumor sections was performed using images provided by the Human Protein Atlas (HPA) at proteinatlas.org. Representative images were selected from regions of the tumor tissue and healthy tissue from pancreas, stomach and liver containing positive staining for the antibody CAB005268.

### Data availability:

Raw data for this study were generated at the University of California, San Diego Moores by the Sandip Patel Lab (https://moorescancercenter.ucsd.edu/research/centers-and-labs/patel-lab/index.html). Derived data supporting the findings of this study are available from the corresponding author upon request.

## Results

### Apoptosis and JUN signaling gene signatures are upregulated within the tumor-nerve microenvironment of KPC mice.

**Figure 1:**
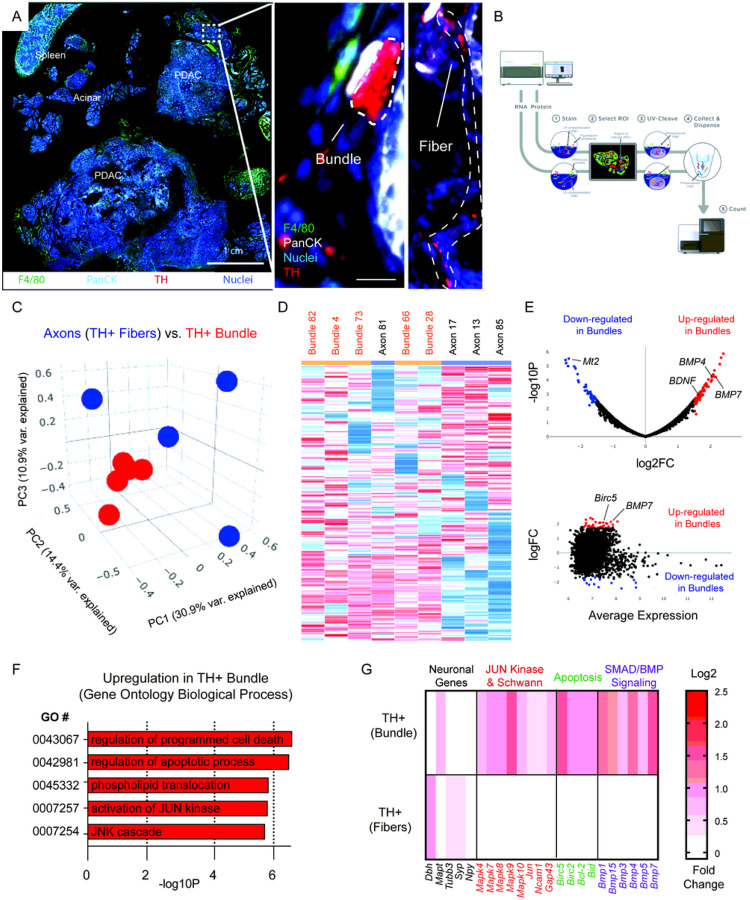
Digital spatial profiling of the tumor-nerve microenvironment in KPC mice reveals transcriptomic upregulation of apoptotic processes and JUN signaling gene signatures. (A) Immunofluorescence of a whole mount tissue section containing PDAC tumor tissue, adjacent acinar tissue and spleen. Nuclei (blue), F4/80 (green), PanCK (teal), tyrosine hydroxylase (TH; red). Scale bar (1 cm, bottom right). High magnification of boxed region highlighted in A showing TH+ axonal fibers, as well as TH+ bundles (red, right panel). (B) Representative scheme of Nanostring’s GeoMx digital spatial analysis pipeline. (C) Principal component analysis of comparing TH+ axonal fibers and larger caliber TH+ bundles. 9 total ROI’s from N = 5 independent bundles and N = 4 axonal fibers. (D) Hierarchical sample clustering based on differential gene expression matrix of transcription pro le on individually isolated TH+ axons fibers and TH+ bundles. (E) Volcano plot and MA plot highlighting significant differential gene expression upregulated (red) and downregulated (blue) in TH+ bundles, as well as the average expression. (F) Gene ontology clustering of the most upregulated pathways based on differential gene expression analysis. (G) Highlighted gene expression changes in gene classes involved in GO processes including JUN kinase, apoptosis, SMAD/BMP signaling. Scale bar log2 fold change in expression.

To investigate tumor-nerve interactions which occur during PDAC, we utilized the KPC genetic mouse model, in which pancreas specific expression of oncogenic Kras and mutant p53 drives development of PDAC. Mice begin to develop pancreatic intraepithelial neoplasia (PanIN) at 8–10wks of age and disease progression to PDAC typically occurs within 16–20wks (Rhim et al., 2013). Once tumors reached 1 cm in diameter, measured by palpation, mice were sacrificed, and immunofluorescence was performed on the frozen fixed tumors using markers for nerves (TH), epithelial cells (PanCK) and macrophages (F4/80). High magni cation images resolved differences in tumor associated nerve structures where axon fibers and larger caliber bundles could be found (Fig. 1A). The labeled cryosections were loaded onto the GeoMx nanostring instrument and a representative diagram of the pipeline is shown (Fig. 1B). Regions of interest (ROI) were collected from the tumor tissue and barcoded by the instrument in a plate for next generation sequence analysis. Principal component analysis was performed on 10 ROIs containing either axon fibers, or larger nerve bundles (Fig. 1C). We found that larger nerve bundles clustered together (red), while axonal ROIs clustered separately (blue). A heatmap was generated from the expression values, which revealed two large distinct clusters which separated the axonal gene signatures from the nerve bundle gene signatures. To note, axon # 81 ROI, clustered more closely with the nerve bundle ROIs (Fig.1D). Differential expression analysis was performed, and a volcano plot (Fig.1E, top) was created to show the most significant upregulated and downregulated genes in nerve bundles, as well as an MA plot to show the amplitude of expression (Fig. 1E, bottom). Amongst the most upregulated genes included BMP4, BMP7 and BDNF. Gene ontology analysis was performed, which revealed upregulation of numerous pathways related to MAPK, JUN kinase, as well as apoptosis gene signatures (Fig. 1F). Based on the GO gene signatures, we selected genes from pathways enriched for apoptosis, JUN kinase, as well as SMAD/BMP signaling, which was highly upregulated in Kegg Pathways (Fig. 1G). Interestingly MT2 was the most downregulated gene in large nerve bundles: it has shown to be highly expressed in healthy nerves, but not in painful neuromas (13), and is lost during chemotherapy induced neuropathic pain (14). These results reveal a unique transcriptional program in cancer associated nerve bundles marked by upregulation of MAPK, apoptosis and JUN gene signatures. This molecular signature has been observed in Schwann cell regeneration, in non-cancer contexts (15, 16). As a check for the fidelity of our analysis, we performed internal controls comparing spleen, acinar tissues (Supplemental Fig. 1) and sequential regions in tumor tissues, compared to non-paired regions (Supplemental Fig. 2). Regions of acinar tissues and spleen showed independent clustering and enrichment for genes involved in pancreatic digestion. Additionally, sequentially matched regions clustered together using PCA analysis. Lastly, the top differentially expressed genes in our data provide insight into tissue type differences that were resolved by nanostring analysis (TH + fibers, bundles, spleen, and acinar) (Supplemental Table 1 and Table 2). Quality control of the data analyzed revealed high quality UMIQ30 values, as well as read correlations with nuclei counts (Supplemental Fig. 3). Taken together, these molecular data indicate a nerve repair program is activated by repair Schwann cells in PDAC associated nerves.

### Nerve associated macrophage express signatures reflective of a role in phagocytosis.

**Figure 2:**
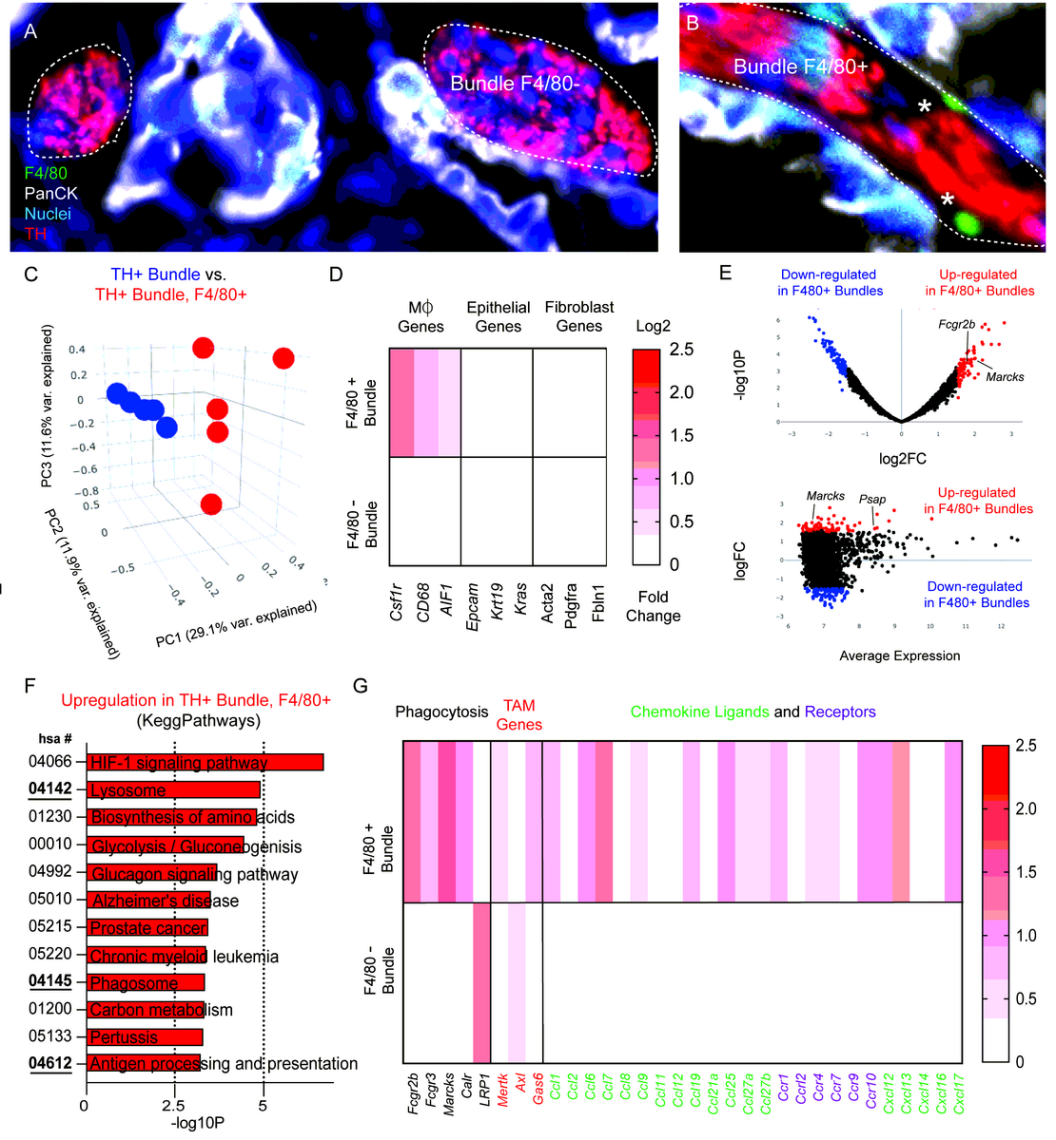
Nerve associated macrophage enriched regions express gene signatures involved in phagocytosis. Image of TH+ nerve bundles selected for nanostring analysis immunostained for macrophages (F4/80+; green), epithelial cells (PanCK; white), nuclei (blue), tyrosine hydroxylase (TH; red) (A) without F4/80- and (B) with F4/80+ labeled cells. (C) Principal component analysis of comparing TH+ bundles and TH+ bundles containing F4/80+ cells. 10 total ROI’s from N = 5 independent bundles and N = 5 bundle + F4/80 ROI’s. (D) Heatmap of differentially expressed genes compared from 10 total ROI’s in F4/80 positive and F4/80 negative TH+ bundles in cell type gene clusters (macrophages, fibroblasts, epithelial). Scale bar represents log2 fold change. (E) Volcano plot and MA plot highlighting significant differential gene expression upregulated (red) and downregulated (blue) in TH+, F4/80+ bundles, as well as the average expression. (F) Gene ontology clustering and KeggPathways analysis of the most upregulated pathways based on differential gene expression analysis. (G) Highlighted gene expression changes in gene classes involved in GO processes including phagocytosis, chemokine ligands and receptors, and TAM genes.

Tissue resident macrophages have unique functions based on their origin and tissue location. For instance, cardiac resident macrophages participate in electoral conduction via gap junction communication with cardiac myocytes (17). While numerous reports have described tumor associated macrophages (TAMs) to function as pro- or anti tumorigenic (18–20), only a few studies have been reported on the function of endoneurial macrophages within PDAC. In these studies, nerve associated macrophages (NAMs) have been shown to secrete neurotrophic factors (21), as well as express in ammatory regulators such as LIF and CTSB (22, 23). However, to our knowledge a molecular characterization of these resident immune cells in-situ has not been performed. Here we performed digital spatial sequencing analysis of PDAC nerve bundles in regions devoid of- or containing F4/80 + macrophages (Fig.2A–2B). Principal component analysis revealed distinct clustering of bundles with and without macrophages (Fig. 2C). Differential expression showed an enrichment for macrophage type associated genes including Csf1r, Cd68 and *AIF1*, enriched in F4/80 + bundles and did not contain upregulated epithelial or fibroblast signatures (Fig. 2D). Macrophage genes reached significance (p = .049 for *Csf1r* and p = .043 for *AIF1*; students t-test). Differential expression analysis was performed, and a volcano plot (Fig. 2E, top) was created to reveal the most significantly upregulated and downregulated genes in nerve bundles, as well as an MA plot to show the amplitude of expression (Fig. 2E, bottom). Amongst the most upregulated genes included Marcks and Fcgr2b, and Psap. Gene ontology analysis revealed upregulation of pathways involved in antigen presentation, phagocytosis and lysosome compartments (Fig. 2F). Based on the GO gene signatures enriched in KeggPathways, we highlighted selected genes from enriched pathways for apoptosis, as well as chemokine ligands/receptors and TAM receptor/ligands, which play pivotal roles in macrophage function (Fig. 2G). We found that CCL7, Cxcl12 and Cxcl13 were amongst the most differentially expressed genes enriched in 5 TH+, F4/80 + ROIs. Interestingly, the aforementioned chemokines have been shown to be important for macrophage chemotaxis to damaged nerves (CCL7) (24, 25), and neuropathic pain (Cxcl12, Cxcl13) (26, 27). To note, many interleukin genes were not included as they were detected at low frequency (Supplemental Table 3). These transcriptomic signatures strongly suggest that local F4/80 + macrophages within nerve bundles have phagocytic function. While their role in PDAC biology has not been described, in the context of nerve injury, nerve associated macrophages have been shown to play a key role in phagocytosis, antigen presentation and clearance of damaged axons during Wallerian degeneration (28).

### Local neuroglia proliferate within the tumor nerve microenvironment.

**Figure 3:**
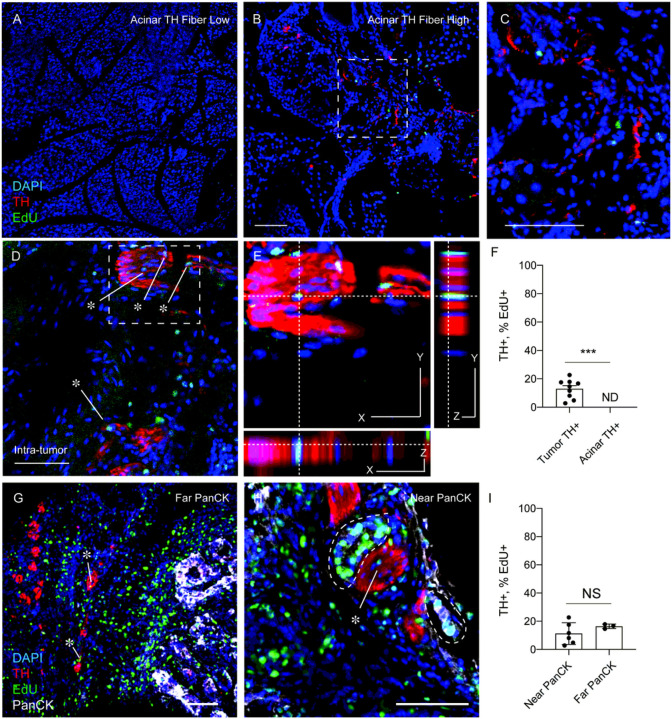
Proliferation of local neuroglia in the tumor nerve microenvironment. (A) Immuno uorescence of KPC mice injected with EdU, sacrificed prior to any tumor development. Pancreas was stained for DAPI (blue), TH (red) and EdU (green). Images show representative regions with low presence of TH+ fibers and (B) TH+ fiber high regions of pancreas tissue. (D-E) Immuno uorescence of nerve bundles from PDAC tumors from KPC mice stained for DAPI (blue), TH (red), EdU (green) and PanCK (white). (E) High magni cations images for colocalization analysis of EdU+ cells within TH+ Bundles. (F) Quanti cation of the density of TH fibers in acinar regions and intra-tumoral regions from KPC mice. N = 9 regions from 3 KPC (G-H) Immunofluorescence of TH nerves, near PanCK epithelial cells (< 50 microns) or far (> 50 microns). Scale bar (50 microns). Unpaired students t-test, *** p-value < .001. (I) Quantification of the percentage of EdU+ percentage of EdU incorporation into cell nuclei within TH+ regions in tumor near PanCK+ or far from PanCK+ cell types. N = 9 total regions from 3 KPC mice stratified into 6 regions near PaNCK+ and 3 not near PanCK+ cell types. Unpaired students t-test was not significant.

Our findings that transcriptomic signatures of non-myelinating repair-type Schwann cells (*BMPs*, *JUN* kinases and *BDNF*) are upregulated in PDAC associated nerve bundles along with nerve-associated macrophage upregulation of phagocytic gene signatures, resembles changes found in damaged nerves during traumatic non-cancer related injuries. In such settings, neuroglial (neural supportive cell types; macrophage, Schwann cells) undergo a functional transformation and proliferate locally to support the repair and regeneration of a damaged nerve (29–31). As such, we hypothesized that during PDAC local neuroglial would actively proliferate at sites of neural damage. To test this, when a palpable tumor was detected, we enrolled mice into daily intraperitoneal injections of EdU (50 mg/kg), which allowed tracking of cell proliferation over multiple days. We performed colocalization analysis in TH + nerves, in acinar regions (remote from the tumor) as well as intra-tumoral TH + nerve bundles. We found in non-tumor tissues, nerve fibers did not colocalize with EdU + nuclei (DAPI) independent of neural density, while in intra-tumoral regions EdU + nuclei were found within TH + bundles (Fig. 3A–3E). Proliferative nuclei were quantified and were significantly increased in TH + regions within tumors compared to TH + regions in acinar tissues, which we did not detect in our analysis (Fig. 3F). These results indicate that neuroglial have functional responses (increased proliferation), based on their proximity to a growing tumor. Given that paracrine signaling factors secreted by the pancreatic cancer TME, such as LIF, SLIT2, and NGF (5, 22, 32), have been shown to promote Schwann cell proliferation in-vitro, we sought to determine whether increased proliferation rates were found in nearby epithelial cells (less than 50 microns), compared to distant TH + bundles (greater than 50 microns). After stratification of intra-tumor TH staining into near epithelial or distant epithelial cells (PanCK+), we did not find a significant difference in TH+, EdU + nerves based on tumor cell location (Fig. 3G–3I). These data indicate that the proliferative phenotype of local neuroglia in the setting of the tumor-nerve microenvironment does not require direct contact with epithelial cells.

### The tumor nerve microenvironment of human PDAC and other gastrointestinal malignancies express markers of non-myelinating Schwann cell phenotypes.

**Figure 4:**
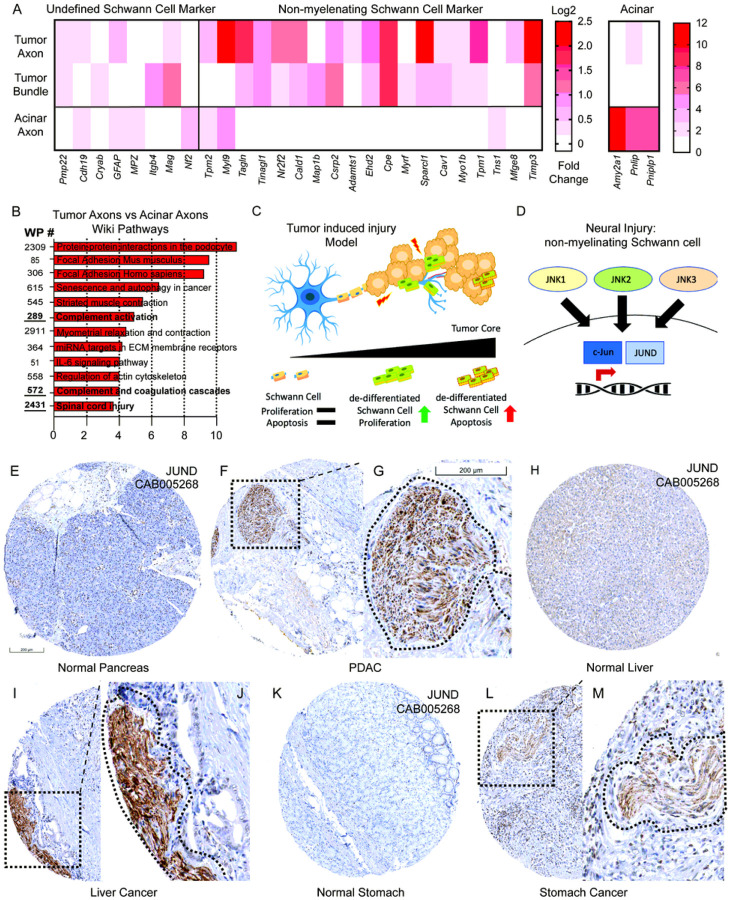
Neural injury and non-myelinating Schwann cell signatures identified in murine transcriptomic analysis are expressed in human PDAC and gastrointestinal tumors. (A) Gene expression analysis of undefined Schwann cell markers, non-myelinating Schwann cells, and acinar genes from TH+ regions in tumor fibers, bundles and acinar regions. (B) Gene ontology analysis using WikiPathways of the most upregulated pathways based on differential gene expression analysis of TH+ fibers in tumor tissues and TH+ fibers in acinar tissue (C-D) Graphical analysis of tumor injury model and non-myelinating Schwann cell transcriptional activation during neural injury. (E-L) Histological analysis of images containing JUND protein signatures from the Human Protein Atlas (HPA) for multiple tumor types and their corresponding healthy organ. (E) normal pancreas (F) PDAC low magnification (G) PDAC high magnification (H) liver (I J) liver cancer (K) stomach (L-M) stomach cancer (Dotted box denotes low magnification while dotted line denotes nerve contour. Scale bar (200 um) in figure E applies to F, H, I, L, Scale bar in figure G applies to J, M.

Given our functional and transcriptomic data in our mouse models, we sought to identify how the transcriptional state of Schwann cells differs in ‘healthy’ acinar tissue and within pancreatic tumors. As we identified transcriptional and functional gene signatures of non-myelinating repair Schwann cells within tumor tissues, we hypothesized that adjacent healthy acinar tissue enriched for TH + axons would have gene signatures of Schwann cells, which did not present a de-differentiated, non-myelinating phenotype. As such, we compared core Schwann single cell transcriptomic signatures de ned by Panglao DB, as well as non-myelinating Schwann cells de ned by OnClass (CL:0002376), and previously published characterized in PDAC (10, 33). In regions containing TH + staining independently of tumor location (acinar, TH + fibers, TH + Bundles), we identified core Schwann cell transcriptomic signatures (*Cryab, MPZ, GFAP*) in all locations, however only in tumor TH + regions (TH + fibers, and bundles), did we identify enriched gene sets characteristic of non-myelinating Schwann cells (15/19 genes in bundles and 17/19 genes in axons) (Fig. 4A). Notably, acinar genes were also highly enriched in acinar regions (Fig. 4A; right panel). No genes were removed from OnClass (CL:0002376) signatures in our comparative analysis, however, 6 genes did not meet the minimum threshold for analysis. Using gene ontology analysis, we compared axons in healthy and tumor regions and identified enrichment scores involved in spinal cord injury and complement pathway activation (Fig. 4B). As these are pathways that occur upon activation of damaged nerves, we hypothesized that a common pathway might be present across multiple tumor models, whereby tumors damage nerves, and promote a non-myelinating Schwann cell transcriptional program (Figs. 4C–4D). To investigate whether this phenotype was present across multiple tumor types, we utilized the human protein atlas to interrogate the tumor-nerve microenvironment across multiple organs and tumor types. Using protein markers c-JUN and JUND which control the repair-Schwann cell phenotype (11, 16), we sought to determine whether these transcription factors were upregulated within PDAC nerve bundles compared to healthy tissues. Using the JUND antibody CAB005628, we identified low protein expression in the normal pancreas (Fig. 4E) while high expression was present in nerve bundles from PDAC (Fig. 4F–4G). These results were highly concordant to nerve bundles found during stomach cancer, and liver cancer (Fig.4H–4L). While, perineural invasion has been reported in many tumor types; reviewed in (34), our data suggest repair-Schwann cells and JUN kinase upregulation in nerve bundles is common across multiple tumor types, and identifies it as a cardinal feature of perineural invasion.

## Discussion

Pancreatic ductal adenocarcinoma (PDAC) has the highest mortality rate of all major cancers and is currently the third leading cause of cancer-related deaths. Survival rates have minimally improved in the last 40 years, with the current five-year survival rate at 11% (35). The failure of current therapeutic approaches has been attributed to many features of PDAC including its inherent drug resistance, poor immunogenicity, and a highly immunosuppressive microenvironment associated with high rates of perineural invasion (PNI; estimated 70–100%) (8, 34). Some emerging strategies for PDAC therapy have focused on altering these physiological features to improve therapeutic efficacy. While perineural invasion is a hallmark of PDAC (36, 37) the etiology and molecular profile of the tumor nerve microenvironment is still not known. Functional studies investigating tumor-nerve interactions began nearly 70 years ago where Levi-Montalcini and colleagues showed that different tumor types were capable of inducing nerve growth. These co-culture experiments led to the discovery of the first neural growth factor NGF, reviewed in (38). More recent studies have utilized co-cultures of dorsal root ganglion with pancreatic cancer cells to reveal effects on cancer cell tumorigenesis (39) invasion (9), as well as growth and proliferation (6, 40). To better understand the native interactions of cells within the complex PDAC tumor microenvironment, an approach that studies a spontaneous intact tumor is advantageous. As such, we utilized digital spatial transcriptomics to characterize PDAC neural bundles in the context of the KPC model and performed proliferation analysis of local Schwann cells in-situ.

Our findings corroborate a recent report which demonstrated that neural bundles found with PDAC tissues are enriched with Schwann cells that have been transcriptionally reprogrammed within the tumor-nerve microenvironment (10, 33). During non-cancer related traumatic nerve injury, genetic signatures (JUN kinase, BMP, MAPK), and functional signatures (neuroglial proliferation, and organization into longitudinal tracks) are indicative of neuronal damage (24, 41, 42). Multiple groups have reported that enlarged neural bundles occur more frequently near the tumor periphery, with fewer bundles occurring in the tumor core (43, 44). Interestingly, we observed proliferation of neuroglia neighboring as well as distant from epithelial cells within the pancreatic tumor. As proliferative neuroglia are a hallmark feature of neuronal injury (11, 29, 45), these data may indicate that physical damage by solid tumor stress might be imparted by cancer cells themselves or the tumor stromal components such as CAFs and the extracellular matrix. Future studies investigating the paracrine chemical (chemokines and neurotrophic factors) and physical (tumor stiffness) features that regulate this process may provide further clarity as to the etiology of perineural invasion.

Notwithstanding, our work has some limitations. While perineural invasion and hypertrophic nerve bundles are found in most cases of PDAC, the tumor-nerve microenvironment is a relatively small physical area (compared to epithelial and stromal components), which requires large tissue fragments to visualize an adequate amount of nerve bundles. To enrich for nerve bundles, we identified KPC tissues with relatively abundant nerve densities to acquire enough cell material for sequencing analysis. Along these lines, the GeoMx digital profiler has tissue size constraints to fit into the imaging window. Due to the large tissue area needed to find nerve bundles, even in KPC mice with substantial PNI, performing digital spatial sequencing on small biopsies containing multiple donors is technically very challenging. While our analyses were performed on sequential KPC tumor slices, to assure the robustness and fidelity of our data we acquired multiple regions of tumor and non-tumor tissues (spleen, acinar), to accurately perform data analysis with appropriate controls. Another limitation is that EdU labeling experiments cannot be replicated in PDAC patients. Given that murine EdU labeling over multiple days results in accumulation of proliferation events compared to single time point at which Ki67 labels cells, we expect only rare proliferation events in humans would be observed. As such human and mouse proliferation data were not compared.

Collectively, our data indicate that peripheral nerve damage occurs during PDAC, findings which are highly concordant with other recent reports (10). Similar transcriptional gene signatures identified in our models were found within human PDAC tissues and other gastrointestinal tumors (JUN signaling pathways). These findings indicate that a common pathway supports the response to neural injury independent of tumor type. While TME induced neural injury appears to underpin a key role in the etiology of hypertrophic nerve bundles, many questions remain regarding the initiating signals of PNI that account for the different rates of PNI across different tumor types (i.e., high in PDAC, while lower in other tumor types) (34). Future studies should investigate where (adjacent acinar, tumor periphery, or tumor core), and whether neurotransmission is disrupted by the growing tumor, as modulation of neural activity via vagal stimulation has been proposed as an experimental approach to the treatment of advanced disease (6, 46). It will also be of interest to study therapies that target specific branches of the autonomic nervous system (sensory, parasympathetic, and sympathetic) as they have been shown to have distinct functions in the TME, and to effect survival in murine PDAC models (47, 48).
